# Phenotypic characteristics and T cell receptor properties in melanoma: deciphering the correlation at single-cell resolution

**DOI:** 10.1038/s41392-021-00864-1

**Published:** 2022-01-04

**Authors:** Yier Lu, Chenyang Ye, Ying Yuan

**Affiliations:** 1 Department of Medical Oncology, the Second Affiliated Hospital of Zhejiang University School of Medicine, Hangzhou, Zhejiang 310009 China; 2 Cancer Institute (Key Laboratory of Cancer Prevention and Intervention, China National Ministry of Education), the Second Affiliated Hospital, School of Medicine, Zhejiang University, Hangzhou, Zhejiang 310009 China

**Keywords:** Tumour immunology, Tumour immunology

The diversity of CD8^+^ tumor-infiltrating lymphocyte (TIL) phenotypes has been described in several cancer types, but the specific relevance remains ambiguous. In a recent article published in Nature, Oliveira et al.^1^ unveiled the relationship between phenotypic properties and specificities of TCRs and they highlighted that the exhausted states are enriched in cells with antitumor reactivity.

TILs have been recently reported to play vital roles in tumor development and immunotherapy efficacy, including adoptive cellular therapy (ACT). Several studies have shown the existence of heterogeneous cellular states of T cells in the tumor microenvironment (TME) and a large number of T cells with nontumor specificity, which largely reduces the effect of immunotherapy.^2,3^ Therefore, as immunotherapy evolves as a feasible strategy for better clinical outcomes, a large effort has been made to discover the features and dynamics of intratumoral and circulating CD8^+^ T cells responding to tumor antigens. Oliveira et al.^1^ correlated the features of T cell responses with the quality and quantity of tumor antigens to characterize the anti-melanoma TCR repertoire; in addition to gaining insights into the TCR repertoire, they uncovered the relationship between the cellular states of TILs and the antigenic specificity of TCRs using single-cell sequencing.

Oliveira et al.^1^ collected five tumor specimens from four melanoma patients and classified CD8^+^ T cells into 13 clusters using high-throughput single-cell sequencing. The subtypes were defined based on the distinction of transcript and surface protein expression, such as TCF7 and IL7R (associated with memory cell states). Although most TCRs mapped to distinct subpopulations, most of the T-cell bearing the same TCRs were restricted to clusters with similar exhausted or memory phenotypes, namely, an: exhausted pattern (T_EX_) or nonexhausted memory pattern (T_NExM_). Thus, Oliveira et al.^1^ hypothesized that this separation is associated with the recognition of different antigens. Oliveira and colleagues also detected antitumor activation of TCRs using multiparametric flow cytometry to screen the upregulation of the CD137 protein, a marker for the measure of reactivity and specificity. Consistent with previous findings, the interaction with tumor antigens within the complex TME may drive CD8^+^ tumor-specific T cells enriched within the T_Ex_ compartment. This bias was also confirmed in the evaluation of blood-derived tumor-specific T cells.

**Fig. 1:**
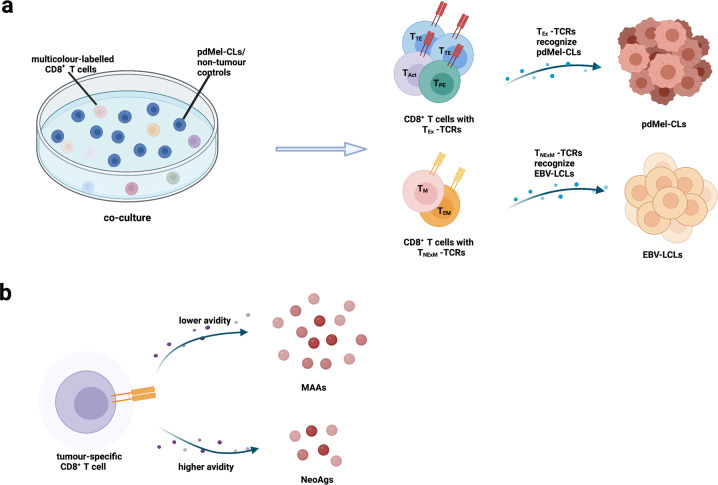
Model of the relationship between cellular phenotype and TCR properties. **a** Measuring upon co-culture of multicolor-labeled CD8^+^ T cells against pdMel-CLs and against EBV-LCLs with peptides from NeoAgs, MAAs or viral antigens. CD8^+^ TILs with exhausted states (including T_TE_, T_Act_ and T_PE_) were enriched in antitumour specificities, whereas CD8^+^ TILs with memory states (including T_EM_ and T_M_) predominantly recognize EBV-LCLs. **b** The concentration level of cognate antigens influences the avidity of tumor specific TCRs. The figure was generated on Biorender.com. Abbreviations: T_TE_ terminally exhausted T cell, T_Act_ acutely activated T cell, T_PE_ precursor exhausted T cell, T_EM_ effector memory T cell, T_M_ memory T cell

To establish a clear connection between TCR properties and tumor recognition, 561 TCRs separated from TILs or blood, were measured upon coculture against autologous Epstein-Barr virus-immortalized lymphoblastoid cell lines (EBV-LCLs) pulsed with peptides including neoantigens (NeoAgs), melanoma-associated antigens (MAAs) and viral antigens. Oliveira et al.^1^ discovered that CD8^+^ TILs, whether with MAA-specific or NeoAg-specific tumor-specific TCRs, preferentially exhibit T_EX_ phenotypes (Fig. 1a). The results suggested that the recognition of tumor antigens potentially leads to the profiles of CD8^+^ tumor specific TILs, whereas the type (MAA and NeoAg) of tumor antigens does not. Notably, CD8^+^ tumor specific TILs bearing Neo-TCRs display markedly higher avidities and lower strength of peptide recognition as their cognate antigens were at substantially lower concentrations, contrasting the majority of MAA-specific TCRs (Fig. 1b). Furthermore, linking the high binding strength of peptide-HLA complexes with the high avidity of NeoAg-TCRs, Oliveira et al.^1^ inferred that the position of the altered residues directly affects the avidity and stability of peptide–HLA complexes to determine the peptide–TCR interaction (Fig. 1b).

While Oliveira et al.^1^ illustrated the correlation of CD8^+^ TIL phenotypes with tumor specificity, Li et al.^3^ identified the dynamic process of CD8^+^ T cell differentiation in the context of the TME in melanoma, and they observed a continuum of exhaustion cell states within the intratumoral T cell pool. In addition, CD8^+^ T cells secrete the CXCL13 protein during their transition from a predysfunctional to a late dysfunctional state, suggesting that this cell population may drive the formation of tertiary lymphoid structures (TLSs) associated with clinical outcomes.^4^ Therefore, further studies need to elucidate their roles in pro-/antitumor immune activity in the different stages of the differentiation trajectory in melanoma. Notably, the presence of CD103^+^CD69^+^CD8^+^ TILs in melanoma, which is associated with a favorable immune response and identified potentially future targets, has been observed.^5^ However, Oliveira et al.^1^ did not divide CD8^+^ TILs into groups characterized by CD103^+^ and/or CD69^+^ through single-cell strategies, in contrast to the former study. The inconsistency according to the marker selected for classification will have to be analyzed and interpreted in future studies, which may elucidate the interaction between CD8^+^ TIL states and tumor antigens in the TME, and boost the effect of immunotherapy.

In conclusion, the study by Oliveira and colleagues represents a significant step toward deciphering mechanisms of the peptide-TCR interaction, consistent with the effect of recognition of tumor-derived antigens. In addition, the indicated data strengthen the basis for the application of TCR-ACT and cancer vaccines for effective and personalized immunotherapy. Thus, the development of bioinformatics technologies for multidimensional analysis targeting T cell signatures should also be taken into consideration.
